# A Prospective Cohort Study Analyzing Radiation-Induced Xerostomia and Quality of Life of Head and Neck Cancer Patients Treated With Intensity-Modulated Radiotherapy and 3D Conformal Radiotherapy Techniques at a Tertiary Cancer Center in Eastern India

**DOI:** 10.7759/cureus.36442

**Published:** 2023-03-20

**Authors:** Bijayalaxmi Sahoo, Sanjukta Padhi, Abinash C Patra, Bikash R Mahapatra, Tanushree Mishra, Saumya Ranjan Mishra, Kanhu Charan Patro

**Affiliations:** 1 Radiation Oncology, Kalinga Institute of Medical Sciences, Bhubaneswar, IND; 2 Radiation Oncology, Acharya Harihar Post Graduate Institute of Cancer, Cuttack, IND; 3 Radiation Oncology, All India Institute of Medical Sciences, Bhubaneswar, Bhubaneswar, IND; 4 Radiation Oncology, Employees' State Insurance (ESI) Hospital, Bhubaneswar, Bhubaneswar, IND; 5 Radiation Oncology, Carcinova Hospital, Cuttack, IND; 6 Radiation Oncology, Mahatma Gandhi Cancer Hospital and Research Institute, Visakhapatnam, IND

**Keywords:** 3d conformal radiotherapy, xerostomia-related quality of life questionnaire, head and neck cancer, parotid scintigraphy, intensity-modulated radiotherapy, xerostomia

## Abstract

Introduction

Cancer of the head and neck is one of the most common cancers in India. Radiotherapy (RT) plays a vital role in the management of head and neck cancer both as a curative and adjuvant modality. Xerostomia is the most common acute and late toxicity. Therefore, this study aimed to analyze radiation-induced xerostomia and the quality of life of patients treated with intensity-modulated radiotherapy (IMRT) and three-dimensional (3D) conformal radiotherapy (3DCRT).

Objectives

We aim to evaluate radiation-induced acute xerostomia both subjectively and objectively at three-month and one-year post-radiotherapy follow-up period in patients who received radiotherapy in conformal technique (IMRT) to the head and neck region and compare it with those who received the 3DCRT technique. We also aim to assess the recovery of salivary flow in the third month post-radiotherapy by measuring the parotid scintigraphy excretion fraction.

Materials and methods

Forty patients with head and neck squamous cell carcinoma (SCC) were randomly assigned to the IMRT and 3DCRT arms. Xerostomia during radiation and at three-month and one-year post-radiotherapy follow-up was assessed subjectively using the xerostomia-related quality of life (XeQOL) questionnaire and objectively by measuring the salivary flow rate and parotid scintigraphy.

Results

The result is analyzed using an independent t-test, Mann-Whitney U test, and Fisher’s exact test. The analysis showed that patients treated with radiation by IMRT showed better XeQOL scores (43.40±2.326 in IMRT and 52.10±2.573 in 3DCRT, p<0.001) and Eating Assessment Tool-10 (EAT-10) score (27.65±2.796 in IMRT and 33.80±1.936 in 3DCRT, p<0.001) compared to those treated with 3DCRT. Analysis of the excretion fraction (EF%) of parotid scintigraphy depicted improvement in EF% for both right and left parotids in the IMRT arm with statistical significance (for right parotid, 25.22±12.98 in IMRT and 19.60±10.17 in 3DCRT, p=0.136, and for left parotid, 28.03±12.51 in IMRT and 15.35±11.49 in 3DCRT, p=0.0019). The mean rate of flow (ROF) of saliva showed a declining trend during the end of radiotherapy treatment compared to baseline, but the mean ROF of saliva was better in IMRT compared to 3DCRT, and the difference was statistically significant. The ROF of saliva starts improving during the one-year post-radiotherapy follow-up period. Pearson’s chi-square test was used to analyze the correlation between mean parotid dose with EF% of parotid scintigraphy, and it showed a negative correlation, which is statistically significant for both 3DCRT and IMRT arms.

Conclusion

Xerostomia can be reduced by precision radiotherapies such as the parotid-sparing IMRT technique in head and neck cancer patients, hence improving the quality of life.

## Introduction

Head and neck cancer are one of the most common cancers in India, comprising almost one-third of the total cancer burden. According to Globocon 2020, 377,713 new oral cavity cases were diagnosed worldwide, constituting 2% of total cases. Out of the above cases, 18,381 new cases were diagnosed in Southeast Asia [1]. The standardized incidence rate for oral cavity cancer is 3.3/100,000 males and 1.8/100,000 females. Multidisciplinary management is essential in most cases, involving surgery, radiotherapy (RT), and chemotherapy. Radiotherapy (RT) plays a vital role in the management of head and neck cancer both as an adjuvant and curative modality. The conventional RT to head and neck cancers typically involves irradiation of major salivary glands and a large area of normal mucosal irradiation. It leads to mucositis, dysphagia, and xerostomia. Xerostomia is the most prevalent acute and late side effect of head and neck malignancy, which leads to decreased quality of life of the patients [2]. Radiation-induced xerostomia outcomes are very variable as per many randomized controlled trials, ranging from acute xerostomia (within three months) to delayed xerostomia (within 1-2 years of the completion of radiotherapy) [3]. The evolution of advanced imaging and conformal radiotherapy techniques such as intensity-modulated radiotherapy (IMRT) allows dose sculpting from critical organs and dose escalation in target regions. Conformal radiation techniques such as intensity-modulated radiotherapy (IMRT) enable a significant reduction of radiation dose to the salivary glands without compromising the dose distribution to the planning target volume (PTV) by modulating the intensity of the radiation beam. The IMRT technique showed a beneficial effect by reducing radiation-induced xerostomia compared to three-dimensional (3D) conformal radiotherapy (3DCRT) [4]. According to various studies, it is very well known that salivary gland function starts recovering in 3-6 months, and it is improved over time [5,6].

We aim to evaluate radiation-induced acute xerostomia both subjectively and objectively at three-month and one-year post-radiotherapy follow-up period in patients who received radiotherapy in conformal technique (IMRT) to the head and neck region and compare it with those who received the 3DCRT technique. We also aim to assess the recovery of salivary flow at three months post-radiotherapy by measuring the parotid scintigraphy excretion fraction.

Therefore, in this prospective cohort study, we analyzed if IMRT decreases the dose to the salivary gland and improves xerostomia-related quality of life. Also, we compare the salivary flow rate by measuring the ejection fraction (EF%) between IMRT and 3DCRT.

## Materials and methods

This study was carried out after obtaining approval from the Institutional Ethics Committee of Acharya Harihar Regional Cancer Center in April 2017 (044-IEC-AHRCC). This study is a single-institutional prospective randomized study conducted at the Department of Radiation Oncology.

Forty patients with head and neck squamous cell carcinoma (SCC) were included between June 2017 and July 2018. The sample size was calculated using a 95% confidence interval with a 5% margin of error. These patients were randomly assigned to the IMRT and 3DCRT arms (1:1 randomization) with 20 patients in each arm.

Patients aged more than 18 years with histologically proven SCC of the head and neck region with good performance status (Eastern Cooperative Oncology Group (ECOG) 0-2) were included in the study. Patients who have received previous radiotherapy or chemotherapy were excluded from the study. We also excluded patients having any prior disease related to the salivary gland such as Sjogren’s syndrome or taking medication that may decrease salivary flow (e.g., tricyclic antidepressants and antipsychotics). All patients were assessed during the course of radiotherapy three months and one year after the completion of treatment.

Objective assessment

**Figure 1:**
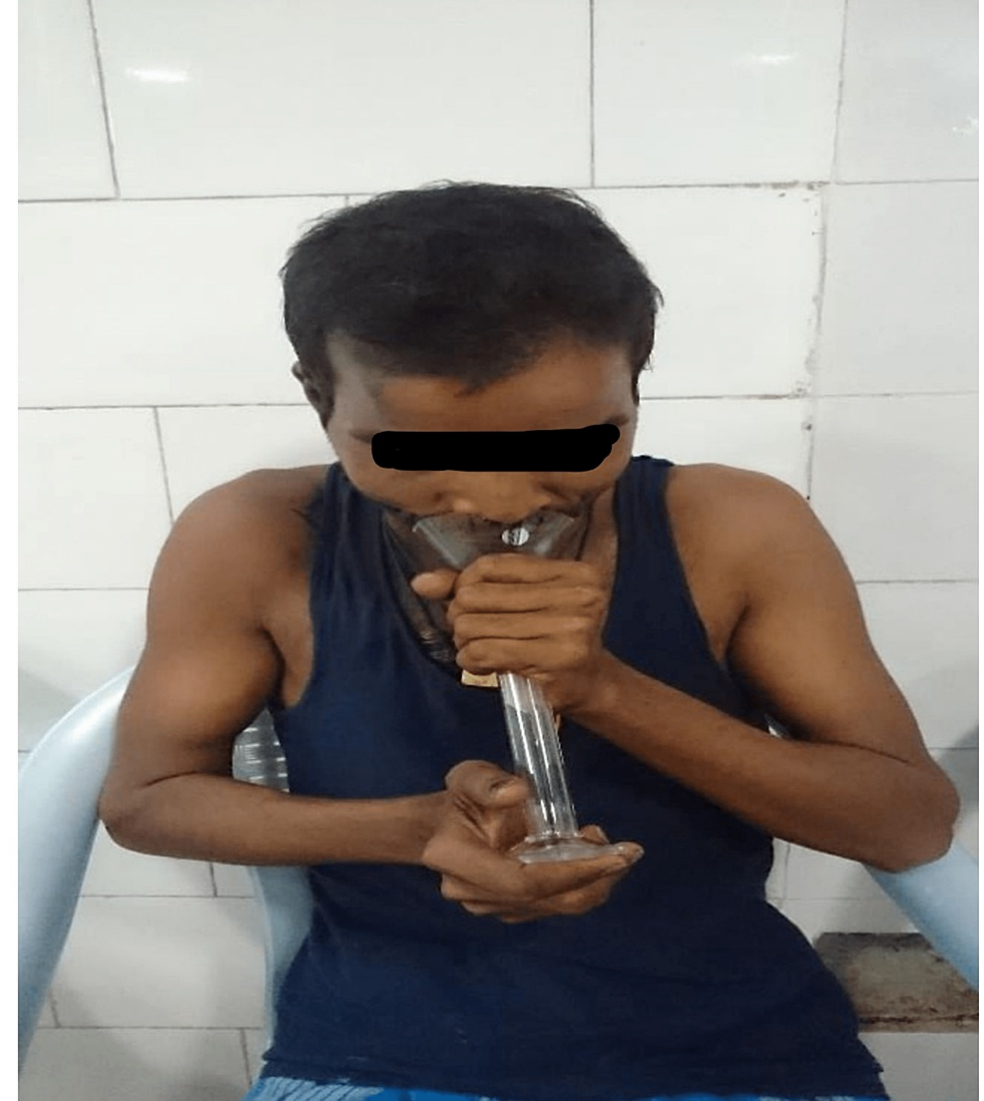
Objective assessment of saliva secretion

The patient was asked to sit in a relaxed stooping forward position. A wide-mouthed glass funnel was given to him/her to spit in for five minutes (Figure 1).

It is connected to a graduated glass test tube below. Water (3 mL) was poured into the test tube through the funnel to collect all saliva that stick into the walls of the funnel and test tube. This process is repeated once at the start of radiotherapy and again at the end of the second week, i.e., during the third week and at the end of the treatment, and then at three-month and one-year post-radiotherapy follow-up.

Salivary scintigraphy scan

**Figure 2:**
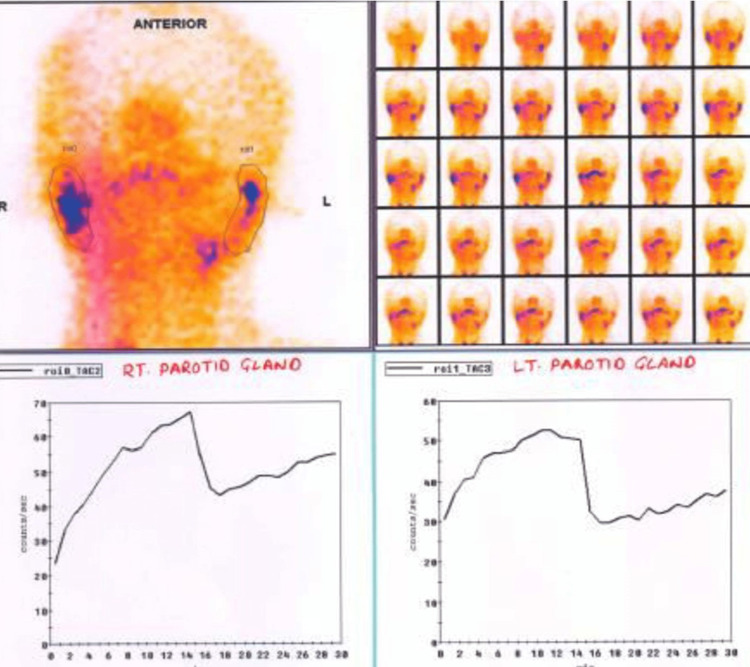
Parotid scintigraphy showing saliva excretion fraction calculation

Scintigraphy scans were performed before the initiation of radiotherapy and at the third month post-radiotherapy. Image acquisition was performed using a gamma camera, equipped with low-energy collimators. Dynamic imaging was performed at a capture rate of one minute/frame for 30 minutes after intravenous injection of 10mCi of Tc99mO4 [7]. After 15 minutes, 5 mL of lemon juice was administered orally as a sialagogue to induce the excretion of saliva. The patient was told to distribute the lemon juice around his mouth and then swallow it. The pre- and post-syringe images were acquired for quantification purposes. Two oval regions of interest (ROIs) were drawn over both parotids. One banana-shaped ROI was also drawn around the right clavicle region for background clearance. Percentage excretion fraction (EF%) was determined using time activity curves (Figure 2).

Qualitative assessment

A questionnaire regarding xerostomia-related quality of life was distributed to all patients during their first week of treatment, in the third week of treatment, after the completion of treatment, and at three-month and one-year post-radiotherapy follow-up. The questionnaire included a demographics section and a xerostomia-related quality of life (XeQOL) questionnaire [8]. A score of 0-4 was assigned to each answer response, which corresponds to the severity of xerostomia to quality of life. There were a total of 15 questions, with a minimum score of 0 and a maximum score of 60. The higher the score, the poorer the quality of life (Table 1).

**Table 1:** XeQOL questionnaire XeQOL: xerostomia-related quality of life

Circle the appropriate response (0 = not at all, 1 = a little, 2 = somewhat, 3 = quite a bit, 4 = very much)					
1. My mouth/throat dryness limits the kinds or amounts of food I eat.	0	1	2	3	4
2. My mouth/throat dryness causes discomfort.	0	1	2	3	4
3. My mouth/throat dryness causes a lot of worry or anxiety.	0	1	2	3	4
4. My mouth/throat dryness impacts my social life.	0	1	2	3	4
5. My mouth/throat dryness makes me uncomfortable when eating in front of other people.	0	1	2	3	4
6. My mouth/throat dryness makes me uncomfortable speaking in front of other people.	0	1	2	3	4
7. My mouth/throat dryness makes me nervous.	0	1	2	3	4
8. My mouth/throat dryness makes me concerned about the looks of my teeth and mouth.	0	1	2	3	4
9. My mouth/throat dryness keeps me from enjoying life.	0	1	2	3	4
10. My mouth/throat dryness interferes with my daily activities.	0	1	2	3	4
11. My mouth/throat dryness interferes with my intimate relationships.	0	1	2	3	4
12. My mouth/throat dryness has a bad effect on tasting food.	0	1	2	3	4
13. My mouth/throat dryness reduces my general happiness with life.	0	1	2	3	4
14. My mouth/throat dryness affects all aspects of my life.	0	1	2	3	4
15. If you were to spend the rest of your life with your mouth/throat dryness just the way it is now, how would you feel about this?	Delighted	Mostly satisfied	Equally satisfied/dissatisfied	Mostly dissatisfied	Terrible

Another set of questions regarding the effect of xerostomia on eating behavior during radiation therapy (Eating Assessment Tool-10 (EAT-10)) was distributed to the patients along with the above. Each question is scored from 0 to 4 based on the severity of the problem (0 = no problem and 4 = severe problem), and the total score was 40 (Table 2).

**Table 2:** EAT-10 questionnaire EAT-10: Eating Assessment Tool-10

Circle the appropriate response (0 = no problem, 4 = severe problem)					
1. My swallowing problem has caused me to lose weight.	0	1	2	3	4
2. My swallowing problems interfere with my ability to go out for meals.	0	1	2	3	4
3. Swallowing liquids takes extra effort.	0	1	2	3	4
4. Swallowing solids takes extra effort.	0	1	2	3	4
5. Swallowing pills takes extra effort.	0	1	2	3	4
6. Swallowing is painful.	0	1	2	3	4
7. The pleasure of eating is affected by my swallowing.	0	1	2	3	4
8. When I swallow, food sticks in my throat.	0	1	2	3	4
9. I cough when I eat.	0	1	2	3	4
10. Swallowing is stressful.	0	1	2	3	4
Total EAT-10 score					

Radiotherapy treatment

After obtaining informed consent, patients were immobilized with the thermoplastic cast in a supine position. Axial CT sections (3 mm) were acquired with intravenous contrast. MRI as an auxiliary image for registration is used in a few patients. Target delineation and segmentations were done as per standard institutional protocol. The dose constraint to both the parotid glands is a mean dose of less than 26 Gy. IMRT planning was performed using the MONACO treatment planning system, and 3DCRT plans were performed using the Oncentra treatment planning system. All 3DCRT patients were treated in the linear accelerator with electronic portal imaging device (EPID)-based setup verification. IMRT patients were also treated in the linear accelerator, and cone beam computed tomography (CBCT) was used for treatment verification.

Concurrent chemotherapy

Single-agent cisplatinum was used as concurrent chemotherapy if indicated. The dosage was 40 mg/square meter weekly. Radiotherapy was usually given within one hour after cisplatinum infusion.

Statistics

For statistical analysis, data were entered into a Microsoft Excel spreadsheet (Microsoft Corporation, Redmond, WA, USA) and then analyzed using the Statistical Package for the Social Sciences (SPSS) version 21 (IBM SPSS Statistics, Armonk, NY, USA). Categorical data (such as age group, gender, site of primary disease, intent of treatment, and use of concurrent CT) between the two groups, i.e., 3DCRT and IMRT, were compared using Fisher’s exact test. For the quantitative data set (interval/ratio), an initial test for normality (Shapiro-Wilk test) was performed for each data set. A parametric test (independent t-test) was used for data sets having normal distribution, and a nonparametric test (Mann-Whitney U test) was used for data sets having non-normal distribution. The change in a particular variable (such as the rate of flow (ROF) of saliva, XeQOL, and EAT-10) over time was tested using the analysis of variance (ANOVA) (Friedman’s ANOVA test). Pearson’s chi-square test was used to analyze the correlation between the mean parotid dose and EF% of parotid scintigraphy. All tests were considered statistically significant if the p-value ≤ 0.05.

## Results

The median age in the 3DCRT and IMRT groups was 50 years and 48 years, respectively. Out of 20 patients in the 3DCRT arm, only six (30%) were females and 14 (70%) were males. Similarly, the number of males and females in the IMRT arm was 13 (65%) and seven (35%), respectively. In the 3DCRT arm, all 20 patients took radiotherapy with adjuvant intent. However, in the IMRT arm, 10 (50%) patients took radiotherapy with adjuvant intent, and the other 10 (50%) patients were with radical intent. Out of 20 patients in each arm, 11 (55%) patients in the 3DCRT arm and 17 (85%) patients in the IMRT arm received concurrent chemotherapy. Patient demographics are shown in Table 3.

**Table 3:** Patient demographics

Age group (years)	3DCRT	IMRT	p-value
<50 years	10	12	0.348
>50 years	10	8	
Mean age	50	48	
Sex			
Male	14	13	0.735
Female	6	7	
Site of disease			
Oral cavity (excluding the tongue)	20	4	<0.001
Tongue	0	9	
Oropharynx	0	3	
Hypopharynx	0	4	
Intent of treatment			
Radical	0	10	<0.001
Adjuvant	20	10	
Concurrent chemotherapy			
Chemotherapy	11	17	0.082
No chemotherapy	9	3	

**Figure 3:**
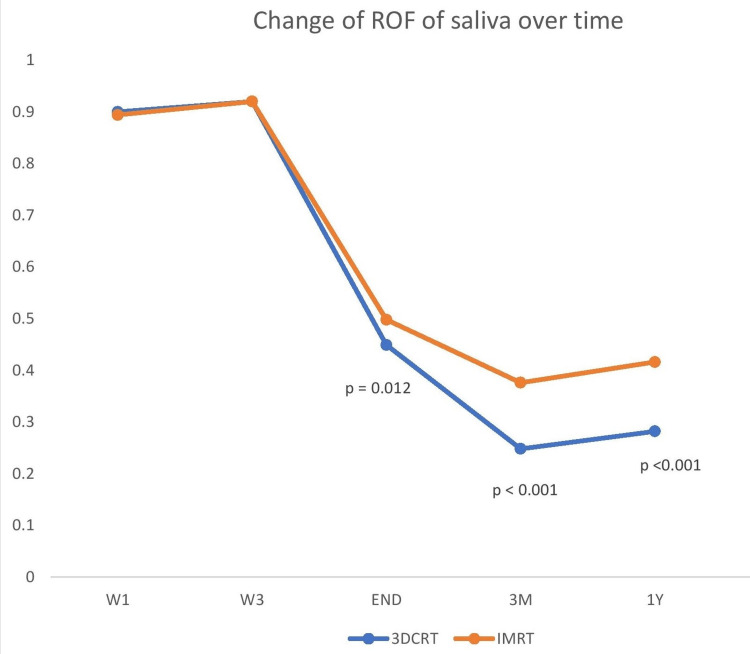
Changes in the ROF of saliva in mL/minute over time between the two arms (3DCRT versus IMRT) ROF: rate of flow, 3DCRT: three-dimensional conformal radiotherapy, IMRT: intensity-modulated radiotherapy

The mean rate of flow (ROF) of saliva at the start of treatment (baseline) in the 3DCRT arm was 0.90 mL/minute and in the IMRT arm was 0.895 mL/minute. At the start of the third week of radiation, the mean ROF of saliva was 0.920 mL/minute for both 3DCRT and IMRT without any statistically significant difference (p=0.904). However, at the end of treatment, there was a statistically significant difference between the mean ROF of saliva in the 3DCRT and IMRT arm (0.449 mL/minute versus 0.498 mL/minute, p=0.012). Similarly, there was a statistically significant difference between the mean ROF of saliva in the 3DCRT and IMRT arm at three months (0.248 mL/minute versus 0.376 mL/minute, p<0.001) and one year post-radiotherapy (0.282 mL/minute versus 0.416 mL/minute, p<0.001). The result was presented in Table 4. The changes in the ROF of saliva over time between the 3DCRT and IMRT arms are demonstrated in Figure 3.

**Table 4:** Comparison of the two groups in terms of the change in the ROF of saliva in mL/minute over time (N=40) ROF: rate of flow, 3DCRT: three-dimensional conformal radiotherapy, IMRT: intensity-modulated radiotherapy, SD: standard deviation, ANOVA: analysis of variance

ROF of saliva	Technique		p-value for the comparison of the two groups at each time point (Mann-Whitney U test)
	3DCRT (mean±SD) (mL/minute)	IMRT (mean±SD) (mL/minute)	
Week 1	0.900±0.072	0.894±0.084	0.841
Week 3	0.920±0.085	0.920±0.096	0.904
End of treatment	0.449±0.049	0.498±0.063	0.012
Three months	0.248±0.051	0.376±0.090	<0.001
One year	0.282±0.055	0.416±0.065	<0.001
p-value for change in the ROF of saliva over time within each group (Friedman’s ANOVA)	<0.001	<0.001	

**Figure 4:**
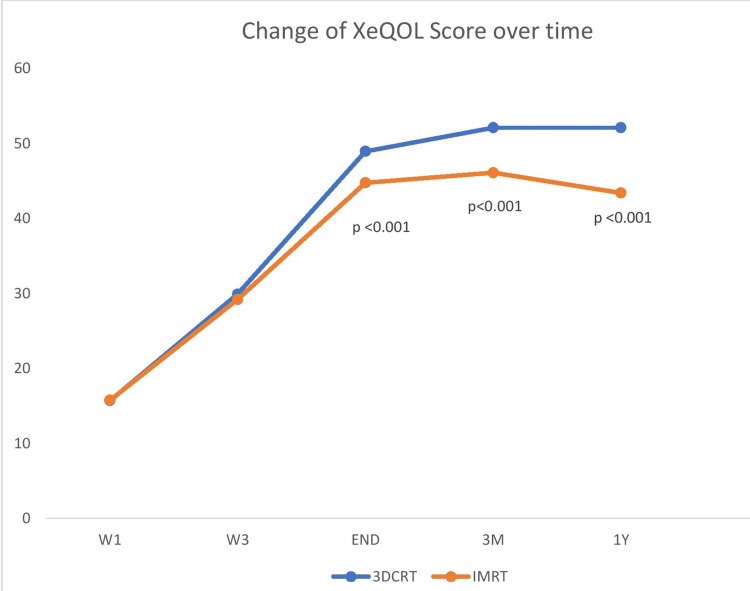
Changes in the XeQOL score over time between the two arms (3DCRT versus IMRT) XeQOL: xerostomia-related quality of life, 3DCRT: three-dimensional conformal radiotherapy, IMRT: intensity-modulated radiotherapy

On objective assessment using the xerostomia-related QOL (XeQOL) questionnaire, the mean score was not statistically different between 3DCRT and IMRT at the start of treatment, i.e., baseline (15.7 versus 15.75, p=0.925) and at the third week of treatment (29.9 versus 29.2, p=0.165). However, a significant difference was found between the two groups (3DCRT and IMRT) as regards the mean XeQOL score at the end of treatment (48.95 versus 44.75, p<0.001), three months after RT (52.1 versus 46.1, p<0.001), and also at one year post-RT (52.1 versus 43.4, p<0.001). This result was depicted in Table 5, and the changes in the XeQOL score over time are shown in Figure 4. This shows that the XeQOL score is better in patients irradiated with the IMRT technique than in those with the 3DCRT technique. After the completion of radiotherapy during the follow-up period, XeQOL improved better with IMRT treatment.

**Table 5:** Comparison of the two groups in terms of the change in XeQOL score over time (N=40) XeQOL: xerostomia-related quality of life, 3DCRT: three-dimensional conformal radiotherapy, IMRT: intensity-modulated radiotherapy, SD: standard deviation, ANOVA: analysis of variance

XeQOL	Technique		p-value for the comparison of the two groups at each time point (Mann-Whitney U test)
	3DCRT (mean±SD)	IMRT (mean±SD)	
Week 1	15.700±1.750	15.750 ±1.831	0.925
Week 3	29.900 ±1.252	29.200±1.542	0.165
End of treatment	48.950±1.431	44.750±2.022	<0.001
Three months	52.100±2.198	46.100±2.174	<0.001
One year	52.100±2.573	43.400±2.326	<0.001
p-value for change in the XeQOL over time within each group (Friedman’s ANOVA)	<0.001	<0.001	

**Figure 5:**
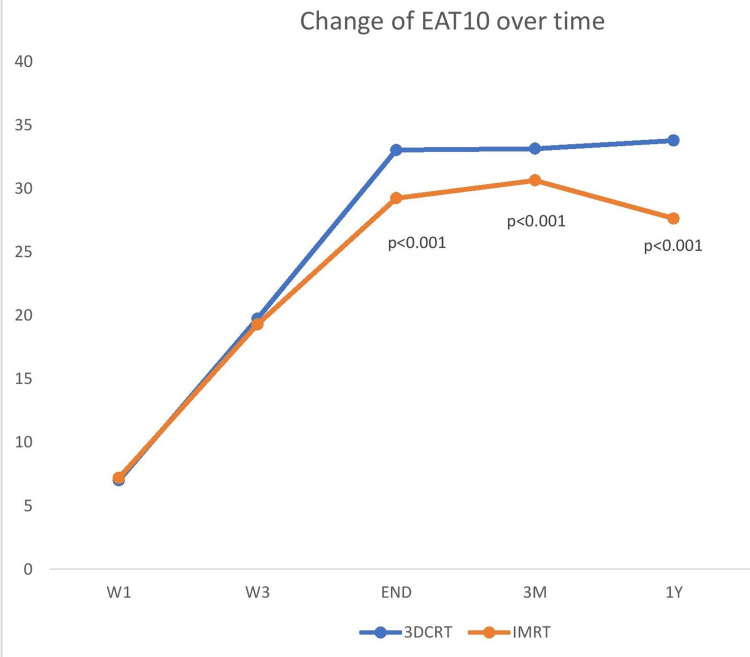
Changes in the EAT-10 score over time between the two arms (3DCRT versus IMRT) EAT-10: Eating Assessment Tool-10, 3DCRT: three-dimensional radiotherapy, IMRT: intensity-modulated radiotherapy

Similarly, for the EAT-10 questionnaire, the mean score was not statistically different between the 3DCRT and IMRT group at baseline (7 versus 7.2, p=0.414) and in the third week of RT (19.75 versus 2.291, p=0.566) and was statistically different at the end of treatment (33.05 versus 30.65, p<0.001), three months post-RT (33.15 versus 30.65, p<0.001), and one year post-RT (33.8 versus 27.65, p<0.001). The result is summarized in Table 6, and the EAT-10 score changes over time were analyzed in Figure 5.

**Table 6:** Comparison of the two groups in terms of the change in the EAT-10 score over time (N=40) EAT-10: Eating Assessment Tool-10, 3DCRT: three-dimensional radiotherapy, IMRT: intensity-modulated radiotherapy, SD: standard deviation, ANOVA: analysis of variance

EAT-10	Technique		p-value for the comparison of the two groups at each time point (Mann-Whitney U test)
	3DCRT (mean±SD)	IMRT (mean±SD)	
Week 1	7.000±0.973	7.200±1.005	0.414
Week 3	19.750±2.291	19.300±2.657	0.565
End of treatment	33.050±2.235	29.250±2.750	<0.001
Three months	33.150±1.899	30.650±2.433	<0.001
One year	33.800±1.936	27.650±2.796	<0.001
p-value for change in EAT-10 over time within each group (Friedman’s ANOVA)	<0.001	<0.001	

The final analysis was done for the percent excretion fraction (EF%) in the parotid scintigraphy scan between baseline and the first follow-up in the third month after the completion of radiotherapy for patients in both the 3DCRT arm and IMRT arm. The patients in the 3DCRT arm had a baseline excretion fraction (EF%) of the right parotid gland of 49.85 and that in the IMRT arm had 51.67 with a p-value of 0.066, which is statistically insignificant. Similarly, patients in the 3DCRT arm had a baseline EF% for the left parotid gland of 26.67 and those in the IMRT arm had 55.30, with a p-value of 0.001. After the third month of follow-up post-radiotherapy, the mean EF% of the right parotid gland in the 3DCRT arm was 19.60 and in the IMRT arm was 25.22, with a p-value of 0.013. Similarly, the EF% of the left parotid gland in the 3DCRT arm was 15.35 and in the IMRT arm was 28.03 with a p-value of 0.0019 (Table 7). The mean parotid excretion fraction percentage of all patients in the 3DCRT arm and IMRT arm showed clinically significant differences, favoring the IMRT arm.

**Table 7:** Comparison of the two groups in terms of the change in parotid scintigraphy excretion fraction over time (N=40) 3DCRT: three-dimensional radiotherapy, IMRT: intensity-modulated radiotherapy, SD: standard deviation

Parotid scintigraphy		Technique		p-value
		3DCRT (mean±SD)	IMRT (mean±SD)	
Right parotid	Week 1	49.85±24.03	51.67±22.08	0.06
	Third-month follow-up	19.60±10.17	25.22±12.98	0.136
Left parotid	Week 1	26.67±23.37	55.30±15.78	0.001
	Third-month follow-up	15.35±11.49	28.03±12.51	0.0019

The mean radiation dose in Gy to the right parotid gland was 37.40±27.95 in the 3DCRT arm and 37.23±11.14 in the IMRT arm, with a p-value of 0.9794, which was statistically not significant. The mean radiation dose for the right parotid does not show any statistically significant result between both arms due to the small sample size for the right-side disease. However, the mean dose in Gy to the left parotid gland was 23.68±28.47 in the 3DCRT arm and 18.64±13.80 in the IMRT arm, with a p-value of 0.00678, which was statistically significant. This showed that the IMRT technique in head and neck cancer causes better dose coverage to target volumes with less dose to the parotid gland.

When comparing the right and left parotid mean doses with parotid scintigraphy results at the third-month follow-up period in 3DCRT patients, it showed a negative correlation with statistically significant results in the left parotid gland. Similarly, IMRT patients showed a negative correlation with statistically significant results in both right and left parotid glands. This is depicted in Table 8.

**Table 8:** Correlation between the mean parotid dose and parotid scintigraphy 3DCRT: three-dimensional radiotherapy, IMRT: intensity-modulated radiotherapy, PS: parotid scintigraphy

Variable	Mean parotid dose (Pearson’s correlation)			
	Right parotid		Left parotid	
	3DCRT	IMRT	3DCRT	IMRT
PS at the third-month follow-up	-0.366	-0.675	-0.718	-0.092
p-value	0.112	0.001	<0.0001	0.0099
Remark	Negative correlation	Negative correlation with statistical significance		

## Discussion

Saliva is a clear slightly acidic mucoserous exocrine secretion. The whole saliva is a complex mix of fluid from major and minor salivary glands [9-11]. The major salivary glands include the paired parotid glands, the submandibular gland, and the sublingual glands. The average daily flow of the whole saliva varies between 1 and 1.5 L. The percentage contributions of the different salivary glands during unstimulated flow are as follows: 20% from the parotid, 65% from the submandibular gland, 7%-8% from the lingual, and less than 10% from numerous minor salivary glands. Stimulated salivary flow changes percentage contributions from each gland, with the parotid contributing more than 50% of the total salivary secretions. The functional cells of salivary glands behave like acute responding tissues to radiation. Radiation-induced xerostomia starts early during treatment [12]. Almost a 50%-60% decline in salivary flow occurs in the first week, and after seven weeks, salivary flow diminishes to approximately 20%. The salivary function continues to decline for up to several months after RT [13]. Thereafter, some recovery is possible until 12-18 months, depending on the dose received by the salivary gland and the volume of the gland included in the irradiation fields [14,15]. Clinically, xerostomia has been reported with as little as two to three doses of 2 Gy. However, doses greater than 30 Gy can cause permanent xerostomia [16,17]. Damage to the salivary glands results in reduced salivary flow, changes in the electrolyte and immunoglobulin composition of saliva, reduction of salivary pH, and repopulation of mouth by cariogenic microflora.

A study by Braam et al. showed a mean stimulated parotid flow rate of 0.29 mL/minute before RT. Six weeks after RT, the mean stimulated parotid flow rate decreased to 0.14 mL/minute (51% reduction), with thereafter an increase to 0.19 mL/minute, 0.19 mL/minute, and 0.26 mL/minute six months, 12 months, and five years after RT, respectively [18]. In the present study, the salivary flow rate began decreasing six weeks from the start of treatment, which further deteriorated during the post-radiotherapy follow-up period. The salivary flow rate is better in the one-year post-radiotherapy follow-up than in the three-month post-radiotherapy follow-up. However, in comparison with the 3DCRT arm, patients taking radiation in the IMRT arm showed a greater saliva flow rate, which is statistically significant.

A study conducted by van Rij et al. concluded that parotid gland-sparing IMRT for head and neck cancer patients improves the xerostomia-related quality of life compared to conventional radiation therapy (CRT) both in rest and during meals [19]. This study used a questionnaire on xerostomia (based on the European Organization for Research and Treatment of Cancer Head and Neck Cancer Quality of Life Questionnaire (EORTC QLQ-H&N35) and an additional trial-specific questionnaire) [8]. Patients treated with IMRT experienced less chewing and swallowing difficulties. They also reported fewer problems with eating and speaking. The overall responses were 85% (n=163), 97% in the IMRT group (n=75) and 77% in the conventional group (n=88). Another study on the effect of IMRT versus CRT on salivary flow and quality of life (QOL) in patients with early-stage nasopharyngeal carcinoma analyzed 51 patients. In the IMRT group, there was a consistent improvement over time with xerostomia-related symptoms significantly less common than in the conventional RT group at 12 months post-radiotherapy [20]. In a prospective longitudinal study of head and neck cancer patients receiving multi-segmental static IMRT, it was derived that after parotid-sparing IMRT, a statistically significant correlation was noted between patient-reported xerostomia and each of the following domains of QOL: eating, communication, pain, and emotion [21]. In our study, xerostomia-related quality of life was better in patients taking IMRT than those taking 3DCRT. The XeQOL starts decreasing from the third week of starting treatment, which further decreases with the increase of radiation doses. However, the XeQOL was better in the IMRT arm compared to the 3DCRT arm on one-year post-radiotherapy follow-up, which is statistically significant.

A study was conducted in the Department of Radiation Oncology, University of Heidelberg, Heidelberg, Germany, to compare changes in salivary gland function after IMRT versus 3DCRT versus CRT with amifostine using quantitative salivary gland scintigraphy (QSGS) [22]. This study concluded that the salivary flow rate began decreasing from the start of the third week of treatment, which further deteriorated with an increased dose of radiation. Goleń et al. conducted a study where pre- and posttreatment salivary excretion fractions (SEFs) were measured in 31 patients treated with IMRT and in nine patients treated with conventional RT. The salivary excretion fraction (SEF) was lower by 52% at six weeks and 35.5% at six months in the conventional arm versus 34% at six weeks and 29.3% at six months in the IMRT arm [23]. An Indian study by Gupta et al. revealed that parotid scintigraphy in 60 head and neck cancer patients treated with 3DCRT and IMRT showed a significant decrease in parotid function even after conformal radiotherapy but to a lesser degree and earlier recovery with IMRT [24]. This study concluded that in the IMRT technique, the median SEF ratios (interquartile range (IQR)) of the parotid glands were 25.7% at three months, 38.2% at 12 months, 59% at 24 months, and 65.3% at 36 months, indicating substantial recovery of salivary function over time, mostly within the first two years of follow-up. The present study also showed that the three-month post-radiotherapy result showed a decrease in parotid scintigraphy result, but the fall in EF% is more in the 3DCRT arm in comparison with the IMR T arm with a statistically significant result.

The study of Xia et al. concluded that inverse planning IMRT causes superior target coverage with the least dose delivery to organs at risk. There was a substantial reduction in the mean parotid dose to as low as 21.4 Gy [25]. Nutting et al., in their parotid-sparing intensity-modulated versus conventional radiotherapy in head and neck cancer (PARSPORT) study, compared conventional radiotherapy with parotid-sparing IMRT in nasopharyngeal carcinoma. At 12 months, xerostomia side effects were reported in 72 patients; grade 2 or worse xerostomia at 12 months was significantly lower in the IMRT group than in the conventional radiotherapy group [26]. The present study also showed similar results as the PARSPORT trial, indicating that IMRT to head and neck cancer showed better dose coverage to target volumes and fewer xerostomia-related complications.

A strong correlation has been shown between the mean parotid dose and residual post-radiotherapy salivary function [27]. There is a gradual decrease in salivary flow with increasing mean parotid dose. Minimal functional impairment occurs at mean doses of <10-15 Gy; increasing doses (in the range of 20-30 Gy) leads to progressive deterioration with severe xerostomia occurring at mean parotid doses of >40 Gy. Roesink et al. reported a significant correlation between SEF ratios and mean parotid doses, both in early (six weeks) and later (one year) follow-ups [28]. Our study also showed a negative correlation between the mean parotid dose with the three-month post-radiotherapy parotid scintigraphy results in both 3DCRT and IMRT arms with statistical significance.

IMRT significantly reduces the incidence and severity of xerostomia compared to 3DCRT in head and neck SCC treated with definitive radiotherapy, with substantial, although partial, recovery of salivary function over time without compromising local control, supporting its widespread adoption in contemporary radiotherapy practice.

Strength of this study

Our study is a prospective randomized study. In our study, we assessed xerostomia subjectively using the XeQOL questionnaire and objectively by measuring both the ROF of saliva and parotid scintigraphy. Very few studies assessed xerostomia by both subjective and objective methods. We also assessed xerostomia for a period of one year, so we were able to assess both acute and subacute toxicity.

Caveats

Our study was a single-institutional study. Larger multicenter studies are required. The number of patients randomized was quite small in each arm. Although the objective assessment of xerostomia by measuring the ROF of saliva was assessed both at the three-month and one-year follow-up period, parotid scintigraphy was assessed only during the three-month follow-up period. The post-radiation xerostomia assessment was during the third month; during this period, some of the acute toxicity may subside. Therefore, the post-radiation follow-up should be the one-month duration for the first three months for the proper assessment of acute xerostomia-related toxicities.

## Conclusions

Both subjective and objective assessments of xerostomia showed better results in IMRT compared to 3DCRT. This single-institutional prospective trial validated that IMRT not only decreases radiation-induced xerostomia but also fastens its recovery. Therefore, it can help in dose escalation to the target without compromising the quality of life. More prospective dosimetric analysis can establish more precise dose-response curves.
